# 阵发性睡眠性血红蛋白尿症诊断与治疗中国指南（2024年版）解读

**DOI:** 10.3760/cma.j.cn121090-20241010-00387

**Published:** 2024-12

**Authors:** 丽燕 李, 蓉 付

**Affiliations:** 天津医科大学总医院，天津市骨髓衰竭及癌性造血克隆防治重点实验室，天津市血液病研究所，天津 300052 Department of Hematology, General Hospital, Tianjin Medical University, Tianjin Key Laboratory of Bone Marrow Failure and Malignant Hemopoietic Clone Control, Tianjin Institute of Hematology, Tianjin 300052, China

## Abstract

阵发性睡眠性血红蛋白尿症（Paroxysmal nocturnal hemoglobinuria，PNH）是一种罕见的后天获得性PIG-A基因突变所致的造血干细胞克隆性疾病，临床主要表现为溶血、骨髓衰竭和高风险并发血栓等，严重时可危及生命。近年来，PNH的发病机制研究及临床诊疗均取得较大进展，中华医学会血液学分会红细胞疾病（贫血）学组在广泛征集专家建议和意见的基础上，结合PNH最新诊治进展、国外相关指南/共识及我国国情共同制订了《阵发性睡眠性血红蛋白尿症诊断与治疗中国指南（2024年版）》。本文将针对PNH诊疗过程中的重点及难点问题进行讨论，对指南的更新部分进行解读，同时结合国内外最新研究进展对指南相关推荐进行拓展，为临床实践提供更多参考。

## Abstract (en)

Paroxysmal nocturnal hemoglobinuria（PNH）is an acquired clonal disorder of hematopoietic stem cells induced by PIG-A gene mutations. It is clinically manifested by hemolysis, bone marrow failure, and high-risk concurrent thrombosis, which are life-threatening in severe cases. Significant progress has been made in the pathogenesis research and clinical diagnosis and treatment of PNH in recent years. The Red Blood Cell Disease（Anemia）Group Chinese Society of Hematology, Chinese Medical Association, combined with the latest diagnosis and treatment progress of PNH, relevant foreign guidelines/consensus, and China's national conditions, jointly formulated the “Guidelines for the diagnosis and management of paroxysmal nocturnal hemoglobinuria（2024）” based on extensive solicitation of expert suggestions and opinions. This article discussed the key and difficult issues in PNH diagnosis and treatment, interpreted the updated part of the guidelines, and expanded the relevant recommendations of the guidelines according to the latest research progress at home and abroad, thereby providing more references for clinical practice.

阵发性睡眠性血红蛋白尿症（Paroxysmal nocturnal hemoglobinuria，PNH）作为一种慢性血管内溶血性疾病，属罕见病范畴[1]。近年来，随着对PNH发病机制的深入探索，PNH临床诊治取得较大进展。本文就中华医学会血液学分会红细胞疾病（贫血）学组发布的《阵发性睡眠性血红蛋白尿症诊断与治疗中国指南（2024年版）》（以下简称“新版指南”）[2]进行解读，为我国血液科医师提供规范化的临床实践指导。

一、PNH克隆筛查

PNH虽属罕见病，发病率低，但因我国人口基数庞大，实际患病人数并不少，且存在诊断难、治疗难和疾病负担重等问题，故PNH诊疗面临诸多挑战。《中国阵发性睡眠性血红蛋白尿症患者生存状况白皮书》（以下简称《白皮书》）调研显示我国PNH患者平均确诊时间为（22.9±36.5）个月，最长确诊时间为227个月；有33.7％的患者曾经历过误诊，平均误诊次数为（1.8±2.4）次。亦有研究结果表明PNH误诊率较高，首次就诊确诊率仅7％，起病1年内确诊者仅35.5％[3]；确诊再生障碍性贫血（AA）、骨髓增生异常综合征（MDS）和不明原因贫血患者，如进行PNH筛查，均有一定比例确诊为PNH[4]–[5]。国外亦有相关研究对PNH克隆筛查进行调研。Spychalska等[6]对波兰35个血液中心的2 790例患者进行外周血PNH克隆检测，结果示322例（11.5％）患者粒细胞检测到PNH克隆，其中40.6％（128/322）具有非常小的克隆或罕见的PNH细胞（≤1％）。在美国队列中，44％的病例通过高灵敏流式细胞术（FCM）检测到非常小的PNH克隆[7]，而西班牙、巴西检出比例较低，分别为10％和16％[8]。故新版指南特别强调PNH克隆的筛查，建议出现以血红蛋白尿为主要表现的血管内溶血（IVH），无法解释的溶血伴有铁缺乏及血细胞减少，外周血成熟红细胞直接抗人球蛋白（Coombs试验）阴性的溶血性贫血等表现均需筛查PNH克隆，还强调不同寻常的血栓形成或出现不能用其他疾病解释的呼吸困难、腹痛、吞咽困难等临床表现，应及时进行PNH克隆检测，避免漏诊。

二、诊断

已有研究证实高达50％～70％的初诊AA患者伴有PNH克隆，但克隆比例较低，该部分患者对免疫抑制剂有较好的治疗反应[9]；调研显示我国PNH患者，经典型PNH占65.3％，合并骨髓衰竭性疾病（BMF）占28.3％，亚临床型占6.4％。因此不能只根据PNH克隆大小直接做出分型诊断，应基于溶血指标、PNH克隆及骨髓细胞学综合判断。新版指南新增PNH临床表现及鉴别诊断，强调在排除先天遗传性疾病后，重点与后天获得性溶血性疾病相鉴别。

FCM可以检测不同血细胞种类GPI锚连蛋白的缺失、量化PNH克隆的大小，同时对PNH微小克隆的检出具有较高灵敏度，成为当前PNH克隆识别及定量的金标准。PNH患者IVH可能使检测到的红细胞PNH克隆比例低于实际水平，因此白细胞PNH克隆的检测尤为重要，高灵敏FCM可检测到0.05％～0.1％的PNH克隆。气单胞菌溶素前体变异体（FLAER）是Alexa-488标记的无活性气单胞菌溶素前体的变异体，诊断PNH更敏感、更特异，可更大程度地避免漏诊和误诊[10]–[11]。Seth等[12]研究结果证实了FLAER和CD157组合在高灵敏度测定中鉴定PNH克隆的稳健性和实用性。故新版指南将FLAER作为PNH诊断的常规检测项目，对粒细胞检测推荐采用FLAER和CD24/CD157，单核细胞采用FLAER和CD14/CD157，当FLAER和上述锚蛋白双阴性时确定为PNH克隆。

三、治疗

（一）总体治疗原则

新版指南强调PNH治疗方案的选择基于疾病临床分型。经典型PNH一线治疗为补体通路抑制剂，目前C5补体抑制剂（依库珠单抗、可伐利单抗）及B因子抑制剂（伊普可泮）均在我国获批治疗新诊断PNH，疗效佳者建议规律用药及随访。疗效不佳者需首先鉴别溶血的性质，针对IVH及血管外溶血（EVH）同时给出治疗建议。亚临床型PNH主要针对BMF进行治疗[13]。合并其他BMF的PNH患者建议应用免疫抑制剂联合促造血治疗，若PNH克隆比例较高且伴有溶血，可应用免疫抑制剂联合补体通路抑制剂；有合适供者的年轻患者可考虑行异基因造血干细胞移植（allo-HSCT）。

（二）补体通路抑制剂治疗

1. 治疗指征及时机：补体通路抑制剂的应用是近年来我国PNH患者治疗取得的最大进展。PNH注册研究（M07-001）评估了依库珠单抗对无输血史和溶血增加相关临床症状［LDH≥1.5正常上限（ULN）］PNH患者的有效性。这些症状被确定为：虚弱、疲劳、血红蛋白尿、腹痛、呼吸困难、贫血（HGB<100 g/L）、重大血管事件（如血栓形成）、吞咽困难或勃起功能障碍。2015年3月，欧洲药品管理局根据上述研究结果定义了PNH的高疾病活动性（High disease activity, HDA）：血清LDH≥1.5 ULN同时存在至少一种或多种上述定义的症状表明存在HDA[14]。新版指南建议凡是粒细胞和（或）单核细胞PNH克隆>10％并伴有严重IVH（即LDH>1.5 ULN）的PNH患者，同时伴有终末器官损害的证据及PNH合并妊娠的患者都是补体抑制剂治疗的适应证[15]–[16]。

2. 补体抑制剂的选择及用法：

（1）远端补体抑制剂：以依库珠单抗为代表的远端补体抑制剂应用于PNH治疗已累积十余年的治疗经验，国外多项临床研究及大型回顾性研究均证实依库珠单抗可减轻慢性溶血、减少输血，显著减少血栓等并发症事件，同时显著改善生活质量，实现生存获益[17]–[19]。2022年依库珠单抗在我国正式获批上市，且于2024年进入国家医保目录，可及性大大提高。新版指南明确指出用药之始应充分告知患者永久性、持续性治疗的必要性。下列情况可考虑停止治疗：①PNH克隆大小随时间逐渐减小始终低于10％；②C5多态性导致依库珠单抗治疗耐药，此时需更换其他非C5补体抑制剂；③allo-HSCT后PNH克隆消失。尽管依库珠单抗疗效肯定，但其治疗局限性也日益凸显：可存在原发耐药、EVH、静脉给药导致患者长期需医院治疗及停药后病情易反复等[20]–[21]。新版指南推荐针对部分患者14 d治疗周期结束时出现的突破性IVH（BTH，即LDH升高和溶血症状的进展），建议可缩短治疗周期或增加剂量[22]。

新型C5补体抑制剂可伐利单抗（crovalimab）作为一种人源化靶向补体蛋白C5的循环单克隆抗体，通过连续单克隆抗体回收技术，结合了等电点、新生儿Fc受体（FcRn）和pH依赖性亲和性技术。这些特点不仅使得它可以与C5高效结合，使内皮细胞对C5的吸收增强、在体内对C5进行处理；也可以使新生儿Fc受体介导的可伐利单抗高效再循环；此外，可伐利单抗的结合位点与目前现有C5疗法不同，可以克服C5多态性，这有可能为特定C5基因突变患者提供有效的治疗选择[23]–[24]。COMMODORE 3是可伐利单抗在中国人群中开展的Ⅲ期、多中心、单臂研究，结果显示可伐利单抗可快速持续控制IVH，维持血红蛋白稳定，降低输血需求，总体耐受性良好，治疗期间BTH发生率低；可4周1次皮下维持给药，提高了给药的便捷性[25]。

尽管C5补体抑制剂开创了PNH靶向治疗新时代，仍有高达88％接受C5补体抑制剂治疗的患者持续性贫血，且一半以上存在输血依赖[26]–[27]。此外，静脉注射、频繁给药等对患者的依从性也存在挑战。

（2）近端补体抑制剂：近年针对补体通路活化的新药研发成为热点。伊普可泮作为补体旁路B因子抑制剂的代表，作用于C5末端通路的上游，不仅可控制PNH的IVH，还可阻止其EVH。2024年欧洲血液学会（EHA）公布了APPOINT-PNH研究（初治PNH患者）48周结果，显示血红蛋白可持续改善，达到或接近正常水平，可显著改善疲劳并长期维持稳定；HGB≥120 g/L的患者比例在第48周高于第24周，避免输血比例高达97.5％。APPLY-PNH研究（经治PNH患者）48周结果显示抗C5转换至伊普可泮队列90％以上的患者都避免输血，疲劳明显缓解并能够维持至研究结束。对于既往接受C5抑制剂治疗（ECU/RAV）疗效不佳的PNH患者，单药治疗可以获得很好的疗效，且具有良好的耐受性和安全性[28]–[29]。因此伊普可泮可能具有较C5单抗更好的治疗优势。

Pegcetacoplan（Empaveli、APL-2）是C3靶向抑制剂，可特异性地与补体C3和C3b结合，从而控制末端补体介导的IVH和C3b介导的EVH，成为首个批准用于成人PNH（美国）、对C5抑制剂反应不足或不耐受（澳大利亚）及C5靶向治疗持续时间超过3个月仍贫血的成人PNH（欧盟）的C3靶向治疗[30]。Pegcetacoplan最初的两项Ⅰb期研究（PADDOCK试验和PHAROAH试验）结果分别证实Pegcetacoplan治疗的有效性和安全性[31]；PRINCE研究结果证实Pegcetacoplan可改善并稳定血红蛋白水平，同时使溶血相关指标（LDH、胆红素和网织红细胞比值）降至正常范围，疲劳评分的功能评估明显改善，安全且耐受性良好，最终可实现依库珠单抗停药。对于稳定但不能使血液学参数正常化的现有C5靶向治疗，Pegcetacoplan有望成为更佳的替代方案[32]–[33]。

3. 感染：补体系统作为机体重要的免疫效应及其链式放大系统，是连接固有免疫和适应性免疫的重要桥梁，在维持机体免疫自稳方面发挥重要作用。补体缺陷者有易感染倾向，常反复发生细菌、真菌感染。经典途径补体成分缺陷（C1～C4）者易感染荚膜菌，旁路途径和终末途径补体成分缺陷者则主要感染奈瑟菌，尤其是对危及生命的脑膜炎奈瑟菌（*N.meningitidis*）的易感性增加。据统计，接受依库珠单抗治疗患者患侵袭性脑膜炎球菌病（IMD）的风险显著增加[34]，大多数病例是由B群和C群病原体引起（分别为69％和22％），B群病原体在美国和欧洲其他地区的患病率也最高[35]。因此，新版指南明确指出所有患者均须在接受补体抑制剂治疗之前至少2周进行疫苗接种，以降低感染风险，且每3年重复接种1次。通过针对性的疫苗接种，IMD感染风险得以明显下降[36]。据统计接受依库珠单抗治疗的患者中，脑膜炎球菌感染的累计报告率在该药物首次获批后随着时间的推移呈下降趋势，并在最近几年保持相对稳定，约为每年0.25/100例患者[16]。

脑膜炎球菌疫苗在妊娠期间的安全性数据相对缺乏，即使仅作为疫情管理的一部分，其风险效益分析也很难评估，因此目前还没有关于妊娠妇女接种IMD疫苗（MenB蛋白疫苗、MenA、MenC和MenACWY蛋白-多糖结合疫苗）安全性的相关研究。目前，脑膜炎球菌疫苗尚未被推荐用于妊娠期。作为预防婴儿疾病的策略，美国疾病控制与预防中心建议妊娠期和哺乳期妇女推迟接种MenB疫苗[37]–[38]。故建议PNH患者妊娠前进行疫苗接种。

虽然建议PNH患者在接受补体抑制剂治疗时采用疫苗接种策略以预防感染，但仍存在突破性感染的可能，可能与下述因素相关：补体失活导致的细菌杀伤受损、菌株覆盖不全及疫苗接种后血清转化的不确定性等。故患者教育和医师关注至关重要。新版指南针对突破性感染的发生给出明确治疗建议，强调抗感染治疗的同时不间断抗补体治疗。

4. EVH：虽然C5补体抑制剂可通过阻断末端补体激活有效控制IVH，但补体替代途径持续激活，C3片段持续在GPI缺陷红细胞上积累，并通过肝脾巨噬系统在血管外被破坏，发生EVH。这可能是部分接受依库珠单抗治疗的患者疗效不佳的重要原因[39]–[40]。新版指南建议若怀疑EVH发生，需完善溶血等相关检查进行鉴别诊断。出现C5补体抑制剂相关EVH，可尝试更换近端补体抑制剂、糖皮质激素等治疗方法。

（三）allo-HSCT治疗

补体通路抑制剂可高效控制IVH，但并不能清除异常的PNH克隆。理论上，allo-HSCT有可能通过调节供者T细胞的细胞毒性和免疫反应性的综合作用来根除PNH克隆，重建正常造血功能，从而实现治愈PNH[41]–[42]。然而PNH作为一种良性克隆性疾病，部分患者生存期仍可在10年以上，且补体抑制剂目前在我国可及性大大提高，可使PNH患者取得与正常人群相当的生存期[3],[43]。allo-HSCT存在植入失败、移植后感染及移植物抗宿主病（GVHD）等风险，影响患者的长期生活质量，甚至危及生命。因此补体抑制剂时代下是否还需要行allo-HSCT是现在的热点问题。针对上述问题，新版指南明确了补体抑制剂时代allo-HSCT治疗的适应证。

结合国外指南的推荐，新版指南推荐了HSCT供者的选择原则，首先推荐移植后造血重建快、并发症少的同胞全相合供者（MSD）。然而我国仅有约30％的患者能找到HLA相合的供者，在青少年及儿童患者中该比例更低。国外已有多项临床研究探讨PNH患者应用无关供者（MUD）和亲缘单倍型供者（HID）HSCT治疗的疗效和安全性，结果发现MSD和MUD移植2年总生存（OS）率和移植物抗宿主无病无失败生存（GFFS）率分别为81.2％和78.1％，差异无统计学意义[44]–[46]。国内亦有多项临床研究[41]–[42],[47]–[48]，分别以HID和MSD为供者进行allo-HSCT治疗PNH，总体造血重建时间、急慢性GVHD发生率、感染发生率、OS、GFFS、移植相关死亡率（TRM）等评价移植效果的关键指标差异均无统计学意义。目前allo-HSCT治疗PNH以MUD和HID作为供者的比例较前明显增加。

allo-HSCT预处理方案主要包括清髓性预处理（MAC）和减低强度预处理（RIC）两类。PNH作为一种良性血液病，首先需确保顺利植入并重建造血功能，同时应尽可能降低患者移植后GVHD的发生率，提高患者的长期生存质量。新版指南针对接受不同供者的预处理方案分别给出治疗建议。期待未来能够进行纳入更多患者的前瞻性研究对可能影响患者预后的因素进行具体研究，确定从HSCT治疗中获益的患者特征，并设计出合理的移植方案，提高HSCT治疗疗效。

（四）PNH并发症处理

PNH并发症的风险评估及相关处理是新版指南的又一亮点内容。新版指南对PNH并发症治疗及管理进行了详细的阐述。此外，新版指南针对PNH合并妊娠及儿童PNH给出了相关治疗建议。

1. 合并血栓：根据国内外研究报道，血栓形成的年化发生率估计为10.61/100例，远高于一般人群[49]。Hill等[50]研究结果表明，与年龄和性别匹配的一般人群相比，合并血栓的PNH患者死亡风险增加13.9倍，10年OS率明显降低，在已知死因病例中，血栓形成占40％～67％。

已有研究证明有血栓形成高危因素且确诊时不伴血栓的PNH患者，不进行与进行华法林一级预防的10年累计静脉血栓发生率分别为36.5％和0％[51]。故新版指南针对未应用补体抑制剂且存在血栓相关危险因素的患者，若没有已知的抗凝禁忌证，可酌情应用低分子肝素或华法林进行一级预防，同时需警惕行一级预防有出血风险，且无法完全控制整体血栓发生率[52]。补体抑制剂通过抑制末端补体活性，降低血管内皮活化；减少凝血酶生成，降低凝血酶-抗凝血酶复合物水平，有效抑制PNH血栓形成并预防血栓复发，血栓年发生率从7.37/100例降低至1.07/100例，显著延长患者生存期[53]，故有血栓形成病史的患者推荐启动“二级预防”，同时继续接受抗血栓治疗以及补体抑制剂治疗，除非存在明确的停药原因（例如出血、重度血小板减少等），防止血栓再次复发[15],[49]。

2. 合并肾功能异常：约64％的PNH通过活检、影像学检查证实患有慢性肾脏疾病，几乎五分之一的患者在筛选时已达到CKD的3～5期[54]。肾衰竭占PNH相关死亡的8％～18％，存在肾功能损伤PNH患者的死亡率是一般人群的7.8倍[55]。Hillmen等[53]研究结果证实补体抑制剂显著降低CKD 3～5期患者的IVH，基线时出现CKD 1～2期的患者在接受补体抑制剂治疗后改善的可能性是恶化的6倍，而CKD 3～5期的改善并不显著。长期使用补体抑制剂治疗后，肾功能改善的可能性增加。Villegas等[56]研究结果证实病程早期给予补体抑制剂治疗可以预防急性肾功能衰竭和进展为慢性肾功能衰竭。故新版指南强调伴肾功能不全患者推荐早期应用补体抑制剂。

3. 特殊人群PNH处理：在补体抑制剂前时代，PNH患者妊娠期间，血栓栓塞并发症及母婴死亡率风险显著增加，PNH孕产妇和胎儿死亡率分别为8％～12％和4％～7％，通常不建议PNH患者妊娠[57]。目前依库珠单抗已获批用于妊娠及哺乳期PNH患者，新版指南建议所有妊娠的PNH患者均需接受依库珠单抗治疗。已有相关研究证实PNH合并妊娠患者接受依库珠单抗治疗可有效控制IVH，降低稳定病情所需的输血频率，对母亲和胎儿是安全的[58]–[59]。尽管产妇死亡率有所降低，但BTH发生率有所上升，故新版指南强调尽管依库珠单抗治疗PNH合并妊娠患者可降低相关风险，仍强烈建议成立包括血液学、妇产科专家及其他相关学科专家的多学科团队共同参与治疗和管理。

（五）疗效标准及随访

新版指南针对PNH免疫抑制剂、HSCT等相关治疗仍沿用上一版共识的疗效标准。目前补体抑制剂在我国可及性大大提高，新版指南新增补体抑制剂治疗相关疗效标准，以期实现国内外评价体系的可比性和一致性。国际相关指南关于补体抑制剂疗效标准的制定源于依库珠单抗关键注册试验相关结果，依库珠单抗的疗效主要基于其对LDH和输血需求的影响，因此该疗效标准将血红蛋白和疾病活动的生物学证据（使用LDH和网织红细胞计数作为溶血的生物标志物）作为补体抑制剂治疗的反应参数[60]。新版指南针对应用补体抑制剂患者的随访给出明确建议，需监测包括溶血、生化、造血原料及PNH克隆等指标，有条件的医院可检测补体活性。

新版指南强调PNH患者随访期间应注重贫血、血栓、感染等相关症状的自我监测，有相关症状发生建议及时就医。针对allo-HSCT患者建议移植后定期监测血细胞分析、溶血相关指标及PNH克隆变化，同时注意监测GVHD、感染症状和动态复查骨髓穿刺等项目。亚临床型及合并其他BMF的PNH患者的随访指标及频率亦给出明确建议，逐一写明，供临床医师参考使用。

四、小结

新版指南进一步规范了我国PNH患者的诊断和治疗，对不同临床类型患者给予不同的治疗方案，以提升我国PNH诊治水平的规范化、精准化和同质化。在中华医学会血液学分会红细胞疾病（贫血）学组的通力协作下，顺利完成了本版指南的撰写，特别感谢全国各中心的参编专家对指南撰写提供的大力支持。
